# Building the future of public health intelligence: updates from the fifth Epidemic Intelligence from Open Sources (EIOS) Global Technical Meeting (GTM) convened by WHO

**DOI:** 10.1186/s12919-025-00340-6

**Published:** 2025-09-15

**Authors:** Raquel Medialdea Carrera, Sami El Sabri, Yasmin Rabiyan, Olga Lugovska, George Sie Williams, Esther Hamblion, Etien Koua, Philip AbdelMalik

**Affiliations:** 1 World Health Organization Hub for Pandemic and Epidemic Intelligence, Berlin, Germany; 2 World Health Organization Regional Office for Africa, Brazzaville, Republic of Congo; 3 World Health Organization, Geneva, Switzerland

**Keywords:** Public health intelligence, Epidemic Intelligence from Open Sources (EIOS), Collaborative surveillance, Artificial Intelligence in public health, Early warning systems, One Health, Workforce development, Health emergency preparedness, Digital surveillance tools, Global health security

## Background and introduction

From December 10 to 12, 2024, the World Health Organization (WHO) convened the fifth Epidemic Intelligence from Open Sources (EIOS) Global Technical Meeting (GTM) in Saly, Senegal. Co-hosted by the WHO Regional Office for Africa (AFRO) and the WHO Hub for Pandemic and Epidemic Intelligence in Berlin and showcasing the newly inaugurated WHO Regional Emergency Hub in Dakar, the 2024 EIOS GTM brought together experts across the globe under the theme, “The future of Public Health Intelligence”.

EIOS is the world’s leading initiative for open-source intelligence in public health decision-making, spearheaded by the WHO and hosted at the WHO Hub for Pandemic and Epidemic Intelligence in Berlin (WHO Hub in Berlin), Germany [1]. The EIOS initiative, established at the end of 2017, brings together new and existing systems, initiatives, and stakeholders to enhance the early detection, communication, and response to public health threats [2]. It leverages artificial intelligence technologies, including machine learning and natural language processing (NLP), for real-time collection and analysis of publicly available information. Built on three pillars – an expanding and well-trained global community of practice, multidisciplinary collaboration, and an evolving, fit-for-purpose system – EIOS provides real-time intelligence to detect and assess public health threats.

Building on the success of previous meetings, this GTM continued to advance the EIOS initiative's goal of creating a unified, all-hazards, One Health approach for early detection, verification, assessment, and communication of public health risks using publicly available information. Over 100 WHO Member States and around 30 organizations participate in the EIOS initiative, and 4500 professionals have been trained in the use of the EIOS system across human, animal, and environmental health sectors over the past 5 years. The initiative plays a critical role in early detection; in the WHO African Region alone, nearly 50% of reported public health events between 2018 and 2023 were detected first through the EIOS system before official notification to WHO [3].

As one of the flagship initiatives of the WHO Hub in Berlin [4], EIOS continues to strengthen capacity and collaboration among and between countries, regional and global actors, to avert and manage public health threats through collaborative surveillance approaches for improved decision-making [5].

### The 2024 EIOS Global Technical Meeting (GTM)

The GTM is the key in-person event for the EIOS community to strengthen networks, address challenges, and build partnerships in public health intelligence (PHI).

Hosting the 2024 GTM in Senegal underscored Africa’s commitment to pioneering PHI, collaborative surveillance and the expansion of innovative surveillance tools. Approximately 200 participants from 70 countries attended the 2024 edition in Senegal, engaging in discussions on PHI advancements, artificial intelligence (AI) applications, workforce development, EIOS-related innovations and collaborative surveillance. The event featured updates on EIOS system improvements and strategies for strengthening global health resilience against emerging threats. The opening ceremony at the WHO Regional Emergency Hub in Dakar (WHO Dakar Hub) included presentations from distinguished speakers:

- Dr. Samba Cor Sarr, on behalf of Dr. Ibrahima Sy, Minister of Health and Social Action of Senegal
- Dr. Chikwe Ihekweazu, Deputy Executive Director of the WHO Health Emergencies Programme
- Dr. Abdou Salam Gueye, Regional Emergency Director, WHO Regional Office for Africa
- Laurent Muschel, Director General, Health Emergency Preparedness and Response Authority of the European Commission (HERA).

A significant milestone highlighted during the 2024 GTM was the continued expansion of the WHO Dakar Hub, inaugurated in December 2023. The WHO Dakar Hub plays a critical role in strengthening Africa’s capacity to rapidly detect and respond to public health threats, with EIOS serving as a key tool in early detection of public health and humanitarian events [6]. The meeting also marked the launch of data.org’s *Epiverse* initiative in Africa, a global collaboration aimed at strengthening epidemic preparedness through trusted data analysis ecosystems, further demonstrating the value of collaborative surveillance and early warning mechanisms in public health intelligence [7].

Throughout the meeting, several key themes emerged, highlighting the importance of building the future of PHI by considering national perspectives, leveraging the potential of AI and technological innovations with a focus on equity, and further building and developing the workforce, operational frameworks and collaborative surveillance. Key takeaways are discussed below.

### Unified efforts in collaborative surveillance for enhanced public health

As countries work to enhance their PHI systems, discussions at the GTM highlighted the need for adaptable surveillance frameworks that align with national structures while fostering strong international and intersectoral collaboration. Countries from different parts of the world shared varying experiences with PHI implementation at multiple levels, highlighting the benefits and challenges of integrating systems like the EIOS system into national structures. While Georgia, Morocco and the Dominican Republic demonstrated how automated media scanning and cross-sector collaboration have improved early detection, challenges around training, limited system interoperability, and sustainability persist. Japan’s experience showed the importance of embedding PHI tools within existing national surveillance networks, while Togo and Thailand emphasized that multisectoral coordination and continuous capacity-building are crucial for ensuring PHI efforts translate into sustainable and actionable intelligence.

Further discussions on collaborative surveillance highlighted that leveraging multiple data sources and fostering cooperation across sectors can significantly improve outbreak detection and response. Case studies from Zambia, Indonesia, and Singapore illustrated how setting epidemiological thresholds, using One Health approaches, and regional data-sharing networks can strengthen decision-making and preparedness at national and regional levels. However, panelists also emphasised that scaling these efforts remains a challenge, particularly in settings with limited resources or political instability and constraints.

The ongoing development of a PHI Operational Framework was a key focus of the 2024 GTM, aiming to harmonize surveillance methodologies and improve coordination across national, regional and global PHI activities. While most countries rely on Event-Based and Indicator-Based Surveillance, inconsistencies in categorization, assessment, and data-sharing protocols continue to hinder evidence-based decision making. In a series of workshops, participants underscored the need for clear PHI terminology, processes, interoperability, and integration of AI to enhance decision-making. Discussions on signal detection, verification, and AI integration reinforced that while technology plays a critical role, success ultimately depends on strong governance, sustainable and skilled workforce capacity, and collaborative decision-making. The lessons learned in these sessions will guide ongoing WHO efforts to refine and develop a flexible PHI framework that is adaptable to regional and country level needs.

### Harnessing AI for public health intelligence: innovation and equity

The discussions on AI highlighted its transformative potential in PHI, with applications ranging from disease outbreak prediction and misinformation tracking to workflow automation and data validation. Real-world examples demonstrated how AI is being used to bridge data gaps, such as forecasting dengue outbreaks in Brazil and Peru, estimating school damage in Gaza using satellite imagery, and analyzing thousands of hours of radio recordings to track misinformation across multiple countries [8]. While AI tools continue to enhance epidemic intelligence, participants and speakers stressed the need for contextualization and adaptability, ensuring that models are tailored to local settings and integrated into existing public health infrastructures, rather than reinventing the wheel. However, better harnessing AI’s potential for PHI was recognized as urgent and essential, requiring sustained investment, cross-sector partnerships, and iterative refinement to enhance accuracy and usability.

A key takeaway was the imperative to make relevant innovations in AI equitable and accessible to ensure that technological advancements benefit diverse populations across the globe rather than exacerbate existing disparities. This issue has long been recognized, but discussions explored concrete initiatives addressing these challenges. Many regions most affected by disease outbreaks also have linguistically underrepresented populations, making it crucial for AI models to support low-resource languages and improve the inclusivity of PHI. Ongoing efforts, such as those of Masakhane and GalsenAI, to bridge these gaps on the African continent were presented, demonstrating how NLP advancements can expand AI’s utility in outbreak-prone settings. Similarly, discussions on predictive modeling initiatives like the Biothreats Emergence, Analysis and Communications Network (BEACON) highlighted the challenge of ensuring real-time forecasting is both accurate and actionable in regions with limited surveillance infrastructure. Presenters emphasized that participatory AI frameworks – involving local communities in data collection and model development – are essential for ensuring AI tools are fair, unbiased, and effective in diverse settings. Participants recognized that while AI offers immense potential for strengthening PHI, its impact will depend on transparent governance, interoperability across systems, and ethical considerations that prioritize sustainability and public good over proprietary interests.

### Collaboration for good is the way forward in public health intelligence

Building on these principles, discussions at the GTM highlighted the critical role of collaboration and community-driven approaches in ensuring that PHI remains inclusive, actionable, and widely accessible. Speakers emphasized that local engagement and participatory surveillance networks are essential for capturing context-specific health insights and fostering trust in public health systems. Initiatives like *Epihack* and *Epicore,* championed by *Ending Pandemics,* demonstrated how bidirectional data collection can empower communities and improve outbreak detection, while the WHO Collaboratory’s digital tools illustrated the potential of global knowledge-sharing networks to enhance analytical capabilities, as demonstrated by the impactful work of the *Mpox analytics* community. Ongoing research in community-driven health monitoring, such as *Influenzanet* and *Respicast*, emphasized the value of leveraging citizen science for real-time surveillance. A key takeaway was that harmonizing data-sharing practices and ensuring equitable access to technological innovations are fundamental to strengthening collaborative surveillance efforts globally.

Technological advancements in PHI were another key focus, with discussions emphasizing that effective innovation relies not only on new tools but also on strong collaborative ecosystems. The upcoming EIOS system v2.0 update was presented as a major step forward in improving collaborative workflows and AI-driven data processing, with new features designed to facilitate real-time information-sharing among public health professionals across borders, sectors and domains. Similarly, projects like UNITE WAVE, which applies AI-powered radio mining in low-internet regions, and FM/AM transcription initiatives in Africa, showcased how technology can increase the reach of PHI exponentially, when implemented in close partnership with local institutions. These initiatives highlighted that technological innovations must be developed in consultation with end-users—including public health agencies, researchers, and local communities—to ensure their relevance, adaptability, and long-term sustainability. Discussions also raised questions about ethics, data ownership, and regional adaptability, reinforcing the need for transparent governance, participatory design, and user-driven contributions in shaping the future of PHI technology.

### Building a sustainable public health intelligence workforce

As technological advancements continue to enhance PHI, the GTM underscored that their impact depends on the ability of a well-trained and sustainable workforce to effectively implement, interpret, and act on these innovations.

Speakers highlighted that lifelong learning and interdisciplinary training are essential to equip professionals with the necessary skills to navigate post-COVID-19 public health challenges. The newly launched WHO Academy presented its vision for scalable, flexible, and personalized learning solutions, emphasizing mentorship, AI-enhanced tools, and communities of practice as critical components for workforce development. Complementing this, data.org’s Capacity Accelerator Network was highlighted as an initiative fostering modular, interdisciplinary learning approaches to build a future-ready PHI workforce at scale.

Similar to the PHI Operational Framework, a key takeaway was the need for a structured yet flexible approach to PHI training that fosters regional expertise while maintaining global standards. Presentations showcased Brazil’s standardized subnational expansion of EIOS system implementation, using innovative training approaches, and Uganda’s multi-sectoral PHI capacity-building program, which was accelerated through a regional EIOS Champions training in Africa. The impact of partnerships was demonstrated through the successful collaboration between the WHO Hub in Berlin, the Robert Koch Institute (RKI) and the WHO Regional Office for the Eastern Mediterranean (EMRO), jointly piloting a PHI capacity building programme in the region. These examples reflect the growing emphasis on tailoring training initiatives to regional, national and subnational contexts. The workshop on PHI training and workforce development further emphasized the need for strengthening capacity at the subnational level, building sustainable evaluation frameworks and designing training packages for different roles, from decision-makers to PHI data managers. The integration of on-the-job learning and mentorship programs to retain expertise and address challenges, such as staff turnover and language barriers, were highlighted as key strategies in this area. Participants also stressed the importance of tailored monitoring and evaluation (M&E) mechanisms such as frameworks or tools to track PHI competencies over time, ensuring that capacity-building efforts result in long-term workforce resilience and improved public health decision-making.

## Conclusions and recommendations

The 2024 EIOS GTM provided a uniquely rich platform for exchanging ideas and experiences among experts from around the world, underscoring the critical importance of PHI in addressing global health challenges. The discussions and presentations highlighted numerous lessons and strategic insights, such as the transformative potential of innovative surveillance tools, AI, capacity strengthening, and community-driven approaches. These insights will inform future planning and shape the future of PHI, while emphasizing the pivotal role of the EIOS initiative in this transformation. Key recommendations include:

- Increase investment in AI and tech innovation. It is essential to enhance early detection, assessment and response by integrating AI into public health systems and promoting equitable access to AI tools.
- Strengthen Collaborative Surveillance at country, regional, and global levels. There is a need to foster multisectoral collaboration at all levels to improve early detection and response to health threats.
- Develop a comprehensive PHI operational framework to empower countries to effectively implement, enhance, and sustain public health intelligence
- Strengthen the PHI workforce. Structured yet flexible approaches to PHI training and mentorship programs to build and retain expertise, especially during health emergencies, are needed.
- Promote PHI community engagement. Local engagement and participation in surveillance networks to capture context-specific health insights and foster trust in public health systems should be encouraged.
- Expand and integrate EIOS training programs. PHI and EIOS system use capabilities within the EIOS community should be enhanced and the training should be integrated into broader PHI capacity building strategies.
- Foster trust through robust public health systems, ensuring effective collaboration and coordination in addressing public health challenges.

In conclusion, the fifth EIOS GTM demonstrated that the future of PHI lies in innovative technologies, fostering trust and collaboration across different sectors, and a sustainable workforce. By embracing these principles, we can build a resilient and inclusive PHI system that effectively addresses global health challenges. The successful conclusion of the 2024 GTM marks a pivotal step forward for the EIOS initiative and provides valuable insights for its Coordination Group and the global PHI community to move forward over the coming years. It sets the stage for continued progress in PHI and collaborative surveillance, reinforcing a global commitment to protecting health and saving lives through innovation and shared expertise.

**Table:** A selection of initiatives and projects presented and discussed at the 2024 EIOS GTM in order of discussion in the meeting:

• WHO Regional Emergency Hub: https://www.afro.who.int/news/senegal-who-launch-regional-emergency-hub-bolster-africas-response-health-crises	
• EIOS Initiative: https://www.who.int/initiatives/eios	
• Epiverse (data.org): https://data.org/news/catalysts-for-change-building-the-future-of-public-health-intelligence/	
• Collaboratory (WHO): https://www.who.int/initiatives/collaboratory#:~:text=Collaboratory%20is%20a%20WHO%20Hub,for%20better%20pandemic%20and%20epidemic	
• UNICEF Office of Innovation: https://www.unicef.org/innovation/	
• Epicore (Ending Pandemics): https://endingpandemics.org/projects/epicore/	
• EpiHack (Ending Pandemics): https://endingpandemics.org/projects/epihack/	
• Influenzanet: https://influenzanet.info/home	
• Respicast (ECDC/ISI): https://www.ecdc.europa.eu/en/publications-data/european-respiratory-diseases-forecasting-hub-respicast	
• Biothreats Emergence, Analysis and Communications Network (BEACON): https://www.bu.edu/ceid/projects-partnerships/beacon/	
• WHO Academy: https://www.who.int/about/who-academy/	
• Strengthening Public Health Intelligence Competencies: https://pandemichub.who.int/	
• Capacity Accelerator Network (data.org): https://data.org/initiatives/capacity/	
• One Health Biosurveillance Framework (Ministry of Health Singapore): https://rr-asia.woah.org/app/uploads/2023/11/singapore-poster-wog-biosurveillance.pdf	
• Multisource Collaborative Surveillance System for Dengue (Ministry of Health Indonesia): https://www.who.int/indonesia/news/detail/30-07-2024-indonesia-takes-decisive--pioneering-action-to-strengthen-multisource-collaborative-surveillance-for-dengue	
The full 2024 EIOS GTM program and Presentations can be viewed on our website here: https://www.who.int/initiatives/eios/eios-global-technical-meeting-2024	

## Data Availability

All slides, presentations, and materials referenced in this manuscript are publicly available on the official meeting website. The full 2024 EIOS GTM program and presentations can be accessed at: https://www.who.int/initiatives/eios/eios-global-technical-meeting-2024.

## Abbreviations

AFRO: WHO Regional Office for Africa
AI: Artificial Intelligence
BEACON: Biothreats Emergence, Analysis and Communications Network
EIOS: Epidemic Intelligence from Open Sources
EMRO: WHO Regional Office for the Eastern Mediterranean
GTM: Global Technical Meeting
HERA: Health Emergency Preparedness and Response Authority of the European Commission
M&E: Monitoring and Evaluation
NLP: Natural Language Processing
PHI: Public Health Intelligence
RKI: Robert Koch Institute
WHO: World Health Organization
